# Esophageal squamous carcinoma in a 25‐year‐old female, changing trend in epidemiology: A case report

**DOI:** 10.1002/ccr3.8134

**Published:** 2023-10-30

**Authors:** Deepika Karki, Prashant Pant, Asim Shrestha, Suchit Thapa Chhetri, Isabel Kadel, Gagan Prakash Karna

**Affiliations:** ^1^ Nepal Medical College Kathmandu Nepal; ^2^ Department of Internal Medicine Star Hospital Lalitpur Nepal; ^3^ Nepalese Army Institute of Health Sciences Kathmandu Nepal

**Keywords:** basaloid squamous cell carcinoma, esophageal carcinoma, p16, young

## Abstract

Timely diagnosis, comprehensive assessment, and a multidisciplinary treatment approach are essential for young patients with esophageal squamous cell carcinoma, even when conventional risk factors are absent. This report emphasizes the need for increased clinical awareness and improved patient outcomes in an evolving epidemiological landscape.

## Graphical abstract

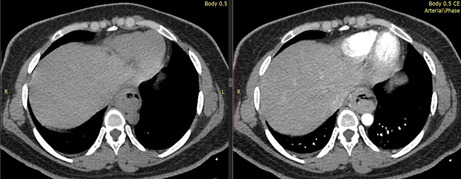

## 1 INTRODUCTION

Esophageal cancer (EC) ranks as the eighth most prevalent form of cancer globally, with squamous cell carcinoma being the predominant subtype.^1^ The mean age of diagnosis of EC is 27–87 years and its occurrence among young people is extremely rare.^2^ Squamous cell carcinoma of the esophagus is characterized by distinct attributes, including squamous dysplasia, nuclear anaplasia, and the presence of keratinocyte‐like cells featuring intracellular bridges or keratinization. This type of carcinoma can manifest as ulcerative or diffusely infiltrative lesions.^3^

Esophageal malignancies are primarily attributed to environmental factors and often develop over a prolonged, latent period of carcinogenesis, contributing to the scarcity of cases among young individuals.^4^ In this context, we present a noteworthy case of squamous cell carcinoma situated in the mid esophagus of a 25‐year‐old woman. This case is notable due to its uncommon occurrence within the younger age group and absence of known risk factors.

## 2 CASE REPORT

A 25 year old female presented with complains of difficulty in swallowing of solid foods for 5–6 months and feeling of food getting stuck at the retro‐sternal region after eating. The dysphagia was progressive, and eventually she had trouble swallowing semi‐solid foods as well. The patient had a history of anorexia and weight loss. There was no record of any prior prolonged medical conditions, previous hospitalizations, or instances of gastrointestinal ailments within the patient's medical history. Furthermore, there was no familial precedent of gastrointestinal diseases or cancers.

### 2.1 Diagnostic assessments

**FIGURE 1:**
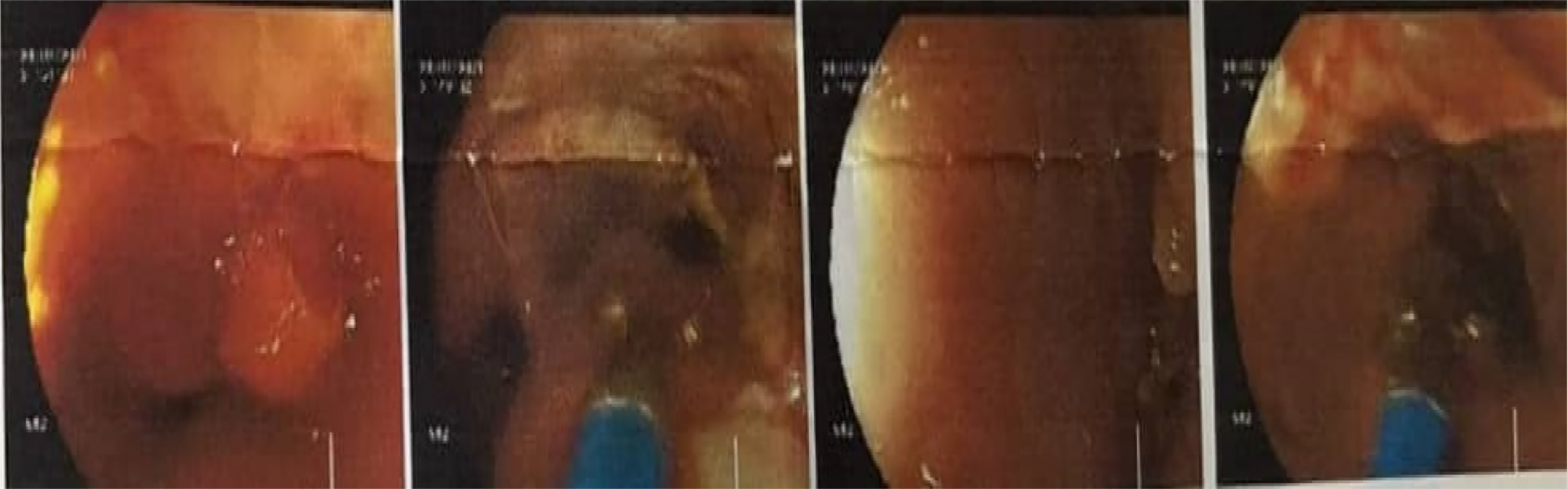
Upper gastrointestinal (UGI) Endoscopy showing Ulcero‐proliferative growth from mid esophagus at 38 cm from incisor.

Physical examinations did not reveal any significant findings, and the laboratory reports showed results within the normal range. An upper gastrointestinal (UGI) endoscopy was performed, revealing the presence of an ulcerative and proliferative growth starting from the mid‐esophagus, located approximately 38 cm from the incisor teeth (Figure 1). This growth extended around the area of the gastro‐esophageal junction. The biopsy taken from the proliferated site exhibited nests of basaloid squamous cells infiltrating the lamina propria, showing mild pleomorphism, oval hyperchromatic nuclei, along with keratin production, and nuclear anaplasia.

**FIGURE 2:**
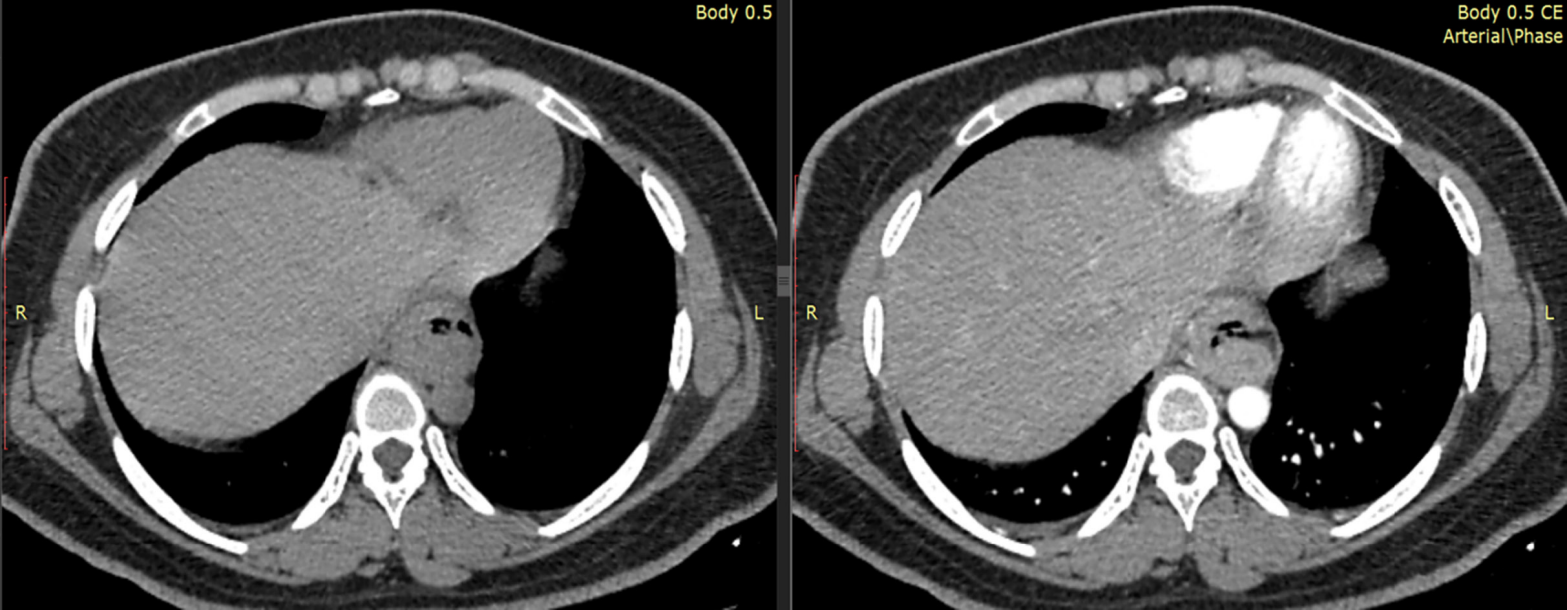
Contrast Enhanced Computed Tomography Scan (CECT‐scan) showing heterogeneously enhancing, diffuse, circumferential, asymmetric thickening of middle and lower thoracic esophagus, extending up to gastro‐esophageal junction, abutting left atrium, descending thoracic aorta and IVC anterior without luminal attenuation/defect.

Afterwards, a Contrast Enhanced Computed Tomography Scan (CECT‐scan) was performed, revealing an 11 cm long segment of middle and lower thoracic esophagus with heterogeneous enhancement and asymmetric thickening of up to 20 mm. These findings were suggestive of a malignant mass (Figure 2). Genetic testing was conducted, indicating that the tumor was positive for p16 and demonstrated proficiency for MMR proteins, specifically microsatellite stability.

### 2.2 Treatment

**FIGURE 3:**
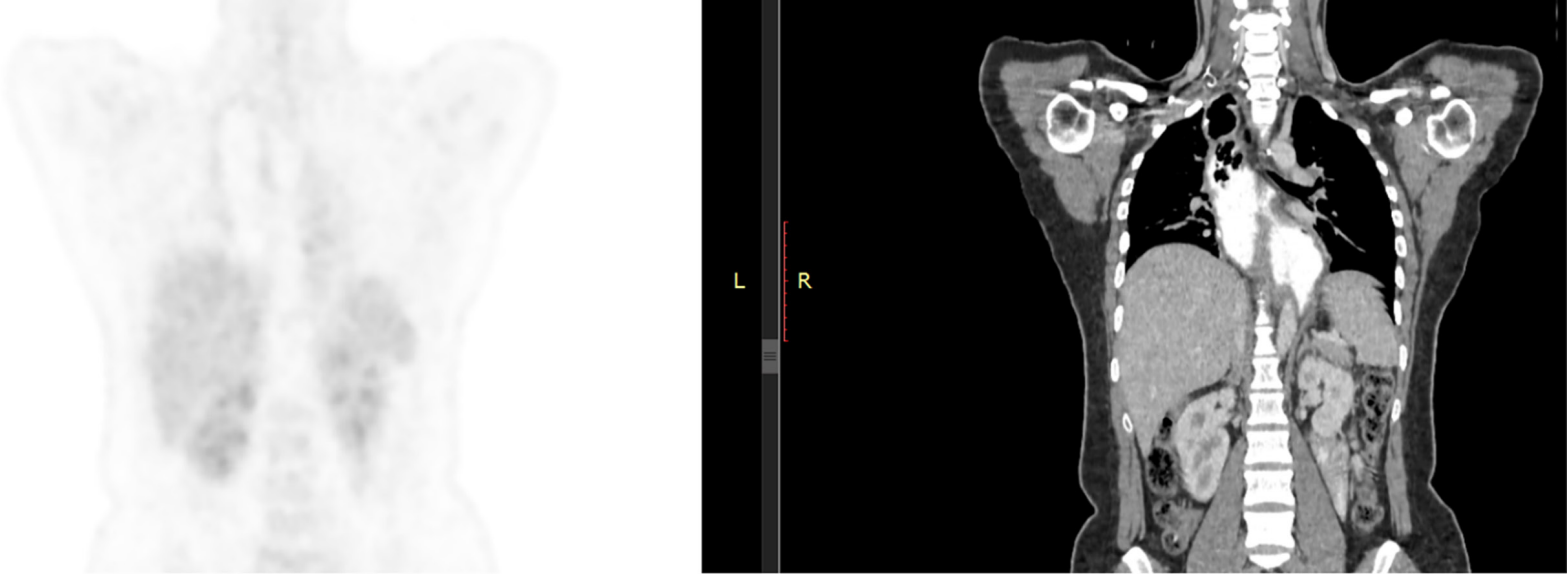
Positive Emission Topography Scan (PET‐scan) showing metabolically active, residual wall thickening, involving lower 1/3rd of esophagus, abutting the adjacent descending thoracic aorta (<90°), without extension into the gastro‐esophageal junction.

The patient was referred to a higher center for further management and investigations. A chemotherapy regimen comprising Inj. Paclitaxel and Inj. Carboplatin was commenced and administered over the course of three sessions. The Positive Emission Topography Scan (PET‐scan) showed metabolically active residual wall thickening in the lower 1/3rd of the esophagus, extending up to approximately 1.4 cm above the gastroesophageal junction. (Figure 3) No metastatic lesions were observed. Radiation therapy for the lower esophagus was administered using the Image‐Guided Radiation Therapy (IGRT) technique, employing 6 MV photons, delivered at a total dose of 41.4 Gy over 23 fractions. This radiation treatment was complemented by four cycles of concurrent chemotherapy consisting of Inj. Paclitaxel (50 mg/m2) and Inj. Carboplatin (AUC 1.5). The irradiation was that of primary tumor location only and no regional lymph nodes were involved.

The follow‐up PET‐scan revealed the absence of metabolically active lesions; however, multiple focal ground glass opacities were detected in the lower lobes of both lungs. Some of these opacities exhibited slight FDG uptake, suggesting a potential infectious or inflammatory nature.

In light of the treating physicians' determination to pursue a more aggressive treatment approach, with the objective of evaluating whether chemotherapy and radiotherapy alone would result in the complete resolution of the pathological lesion, a post‐treatment endoscopy revealed the presence of a minute, barely measurable polyp. Given the patient's youthful age and the desire to prevent potential future recurrence, the surgical team considered completing the tumor resection.

**FIGURE 4:**
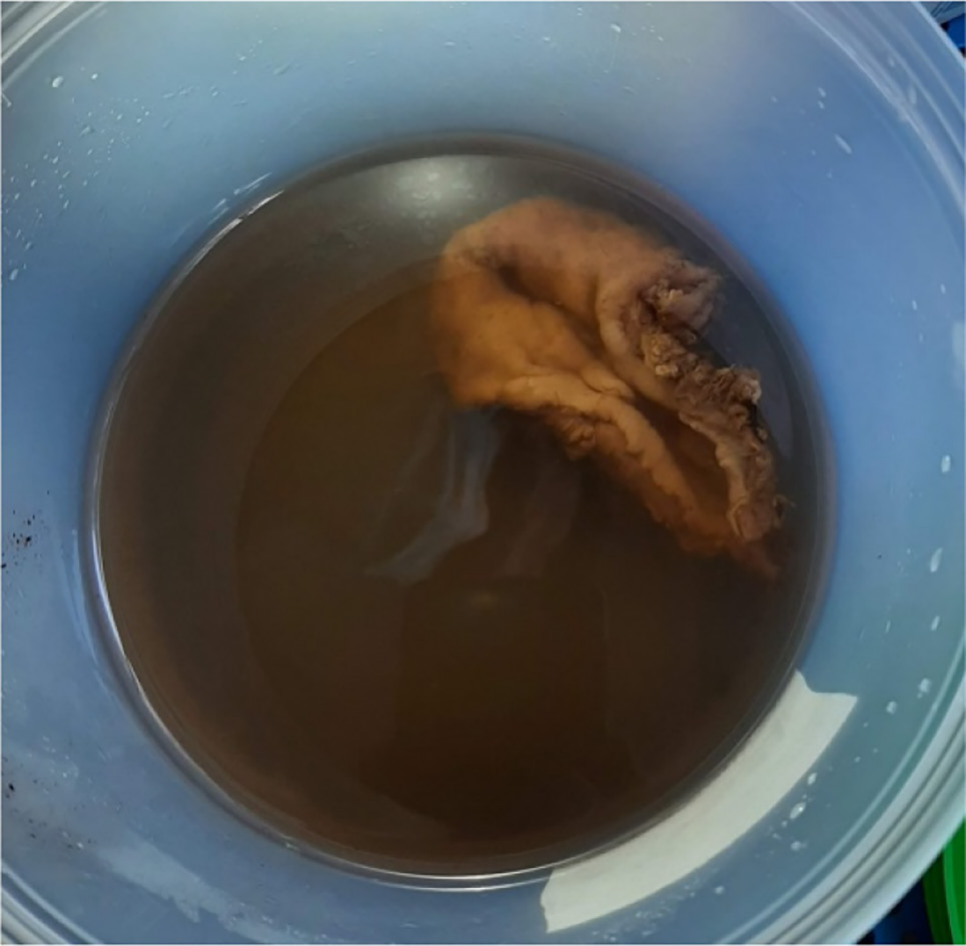
Resected esophageal specimen.

Surgery was scheduled for 4 months following the completion of the last radiotherapy session. The patient underwent a Video‐assisted Thoracoscopic Surgery (VATS) and esophagectomy. For a week after the surgery, she was maintained on nil per oral status, receiving nutrition through a jejunostomy tube and a nasogastric tube was inserted. A week after the surgery, small feeds were initiated through the nasogastric tube, which was subsequently removed, and the patient started taking small amounts of food by mouth. The excised esophagus, along with 13 lymph nodes, was examined for metastasis (Figure 4). The evaluation revealed a polypoidal structure lined by stratified squamous epithelium with underlying fibrous tissue. No residual tumor cells were observed, and the resected margins at both the proximal and distal ends were clear of cancer cells.

### 2.3 Follow‐up

During the initial follow‐up visit, which occurred 1 month after the surgery, a comprehensive assessment was conducted, including hematological and biochemical tests, a full blood checkup, ultrasound, and x‐ray. These evaluations showed no abnormalities. At that point, the feeding jejunostomy was removed, allowing her to gradually transition to a diet of semisolid food approximately 1 month after the surgery. Currently, at the 6‐month mark post‐surgery, she continues to be under continuous follow‐up.

However, she has been experiencing complications such as hoarseness of voice, voice fatigue, frequent episodes of vomiting, and a decrease in appetite. It is reassuring that she has not exhibited symptoms indicative of recurrence or metastatic disease. The combination of an early diagnosis, aggressive treatment, and her relatively young age has contributed to a favorable prognosis and her swift recovery. Regular follow‐up and management will be essential to address the existing complications and ensure her ongoing well‐being.

## 3 DISCUSSION

EC is ranked eighth in global cancer prevalence and is the sixth leading cause of cancer‐related deaths, particularly prominent in developing countries where over 80% of such deaths occur with the squamous variant being more prevalent than adenocarcinoma.^1^ Moreover, the risk of developing the disease elevates beyond the age of 40, increasing with each subsequent decade of life and peaking in the seventh decade. Additionally, its incidence is three times higher in black individuals compared to whites.^5^

In many endemic regions, the squamous variant of this carcinoma is notably influenced by environmental factors, and along with nutritional deficiencies and the consumption of carcinogenic‐laden spices, are major risk factors.^6^ Chronic use of alcohol and tobacco smoking are particularly significant, with tobacco smoking identified as the foremost risk factor by the International Agency for Research on Cancer (IARC). Current smokers have an escalated risk of three to seven times when contrasted with non‐smokers.^7^, ^8^ Additionally, the risk of EC gradually escalates from light to moderate to heavy alcohol consumption in comparison to non‐drinkers.^9^ While genetic susceptibility can contribute to the development of esophageal carcinoma, family history often does not strongly correlate with the risk of the disease.^10^

TP53 mutations, found in ESCC as well as other cancers, have been identified. A recent study focusing on molecular signatures revealed that mutations in TP53, CCND1, SOX2, TP63, PIK3CA, PTEN, NFE2L2, MLL2, ZNF750, NOTCH1, FGFR1, and RB1 were notably enriched in ESCC.^11^ Additionally, loss‐of‐function germline mutations in BRCA2 were linked to an increased risk.^11^

The foremost and most commonly observed initial symptom is progressive dysphagia, initially pertaining to solid foods and later extending to both solid and liquid foods.^12^ Pain while swallowing is less frequent in the early stages but tends to worsen over time. Considerable weight loss and undernutrition may be evident, especially in cases of esophageal squamous cell carcinoma (ESCC), often due to reduced appetite.

Although computed tomography (CT) scans are commonly accessible and used for imaging, endoscopic ultrasound (EUS) is presently considered a superior imaging modality for accurately staging the tumor (T stage) in comparison to CT scans.^13^ EUS assists in tailoring treatment approaches based on the disease stage, thus influencing overall prognosis. However, EUS does have its own limitations. For assessing the metastatic stage (M stage) of EC, CECT, and FDG‐PET are the preferred imaging methods.^14^, ^15^ Among these, FDG‐PET demonstrates higher accuracy in detecting distant metastasis. Several tumor markers, including H2 RLN, WDR66, and PLA2G2A, have been investigated in relation to ESCC.^16^, ^17^ These markers hold potential for aiding in the assessment of ESCC prognosis.

Surgical resection stands as the primary curative approach for early‐stage EC. When combined with chemoradiotherapy in the adjuvant or neoadjuvant setting, survival rates have shown improvement.^18^ The two frequently performed surgical techniques are transhiatal esophagectomy and transthoracic esophagectomy, utilizing the Ivor Lewis method (also known as the McKeown modification, 3‐hole approach).^19^ The choice between these methods is determined by the tumor's location and size. Chemotherapy options include paclitaxel/carboplatin, cisplatin/fluoropyrimidine, and oxaliplatin/fluorouracil regimens.^20^ For radiotherapy, a recommended radiation dose ranges from 41.4 to 50.4 Gy. A range of non‐surgical interventions, encompassing endoscopic treatments such as endoscopic mucosal resection (EMR), endoscopic submucosal dissection (ESD), and ablative therapies, radiotherapy and chemotherapy have roles in ESCC management.

The paramount and pivotal prognostic factor has been identified as the pathological response to multimodality neoadjuvant concurrent chemoradiotherapy (CCRT). In cases of non‐responder esophageal squamous carcinoma, postoperative adjuvant therapy has been shown to reduce the duration of hospitalization and additionally enhance disease‐free survival rates.^20^

Notably, our patient exhibited none of the aforementioned risk factors associated with the disease's development, nor did her family members have a history of esophageal carcinoma or other types of cancer. The patient underwent neoadjuvant chemoradiotherapy, which yielded a remarkable response. Subsequently, VATS and esophagectomy were performed after establishing her suitability for surgery.

The incidence of EC in individuals aged 30 or younger is a rare occurrence on a global scale. Notably, in the extensive case series reported by Paymaster et al, young EC patients constituted just approximately 1% of their total cases.^21^ The demographic and tumor characteristics of EC in young patients closely resemble those in older patients within the same populations. In cases reported from developing countries, the male‐to‐female (M:F) ratio among young patients was nearly equal at 1.8:1, while in cases from developed countries, it skewed higher at 2.5:1.^22^ Additionally, the majority of young EC patients in developing countries presented with ESCC, accounting for 78% of cases, while in developed countries, ESCC was less common, and comprising only 20% of cases. A case series of esophageal carcinoma in young ages reported that 80% of patients had a family history of cancer, with approximately 43% specifically indicating a family history of EC.^22^ The substantial prevalence of cases reporting a familial history of EC and the apparent concentration within a particular ethnic group raise the possibility of genetic factors playing a significant role in the development of EC in this region. However, it is essential to consider that these observations may also be influenced by shared environmental risk factors, such as socioeconomic conditions, dietary practices, the use of similar traditional medicines, or dietary choices, as well as the prevalence of communicable diseases.^23^

Despite advancements in the identification and treatment of EC, a 5‐year survival rate of 15–20% persists among patients diagnosed with the condition. This underscores the ongoing necessity for palliative therapy in cases of advanced disease. Additionally, there appears to be a shifting trend in the disease's epidemiology, affecting even younger generations devoid of recognized risk factors. This emphasizes the urgency of conducting thorough assessments and pursuing biomarker research to ensure comprehensive disease‐free outcomes.

## 4 CONCLUSION

The case report presented underscores the significance of timely diagnosis and intervention to avert the manifestation of advanced‐stage cases, especially among young individuals. The successful neoadjuvant chemotherapy, radiotherapy, and surgery highlight the potential for favorable outcomes, even in unique cases.

## AUTHOR CONTRIBUTIONS

**Deepika Karki:** Conceptualization; data curation; formal analysis; supervision; validation; visualization; writing – original draft; writing – review and editing. **Prashant Pant:** Conceptualization; data curation; formal analysis; supervision; validation; visualization; writing – original draft; writing – review and editing. **Asim Shrestha:** Conceptualization; data curation; formal analysis; supervision; validation; visualization; writing – original draft; writing – review and editing. **Suchit Thapa Chhetri:** Conceptualization; data curation; formal analysis; supervision; validation; visualization; writing – original draft; writing – review and editing. **Isabel Kadel:** Formal analysis; supervision; validation; visualization; writing – original draft; writing – review and editing. **Gagan Prakash Karna:** Supervision; validation; visualization; writing – original draft; writing – review and editing.

## FUNDING INFORMATION

This article did not receive any grants.

## CONFLICT OF INTEREST STATEMENT

The authors declare no conflicts of interest.

## CONSENT

Written informed consent form was obtained from the patient to publish this report in accordance with the journal's consent policy.

## PATIENT PERSPECTIVE

The patient and her family members were anxious about the patient's condition. They were properly counseled and assured that she would get better.

## Data Availability

All the findings are present within the manuscript.
